# Clinically validated graphical approaches identify hepatosplenic multimorbidity in individuals at risk of schistosomiasis

**DOI:** 10.1098/rsos.242256

**Published:** 2025-07-16

**Authors:** Yin-Cong Zhi, Simon Mpooya, Narcis B. Kabatereine, Betty Nabatte, Christopher K. Opio, Goylette F. Chami

**Affiliations:** ^1^ Big Data Institute, Nuffield Department of Population Health, Oxford, UK; ^2^ Republic of Uganda Ministry of Health, Kampala, Central Region, Uganda; ^3^ The Aga Khan University Hospital Nairobi, Nairobi, Nairobi County, Kenya

**Keywords:** complex network, schistosomiasis, portal hypertension, threshold, graph neural network, hepatosplenic

## Abstract

The global burden of multimorbidity is increasing yet poorly understood, owing to insufficient methods for modelling complex systems of conditions. In particular, hepatosplenic multimorbidity has been inadequately investigated. From 17 January to 16 February 2023, we examined 3186 individuals aged 5–92 years from 52 villages across Uganda within the SchistoTrack Cohort. Point-of-care B-mode ultrasound was used to assess 45 hepatosplenic conditions within the context of schistosomiasis (*Schistosoma mansoni*). Three graph learning methods for representing hepatosplenic multimorbidity were compared. Thresholds for including graph edges were found using graph kernels and tested with graph neural networks to assess predictive utility for unobserved conditions. Clinical validity was assessed by identifying medically relevant condition inter-dependencies for portal hypertension. 54.65% (1741/3186) of individuals were multimorbid with two or more hepatosplenic conditions. Thresholds were 50.16 and 64.46% for graphical lasso and signed distance correlation, respectively, but could not be inferred for co-occurrence. Co-occurrence graphs were clinically uninformative with low predictive capacity. Graph learning algorithms with statistical assumptions, e.g. graphical lasso, enabled accurate and clinically valid multimorbidity representations. Severe conditions related to portal hypertension were predicted with high sensitivity and specificity. This work presents a generalizable framework for understanding multimorbidity to enable more accurate diagnoses of complex diseases.

## 1. Introduction

The burden of multimorbidity is growing worldwide with an estimated pooled prevalence of at least 33% across high- and low-middle-income countries [1,2]. Multimorbidity is defined as the co-occurrence of two or more chronic health conditions within an individual. Individuals with multimorbidity often are of low socioeconomic status, have greater number of years lived with disability, and experience early mortality [3]. Hepatosplenic diseases are a particularly complex multimorbidity problem in sub-Saharan Africa with diverse causes ranging from infectious pathogens to non-communicable aetiologies [4]. There are unique challenges posed by multimorbidity to conventional medical curricula and constrained health systems that cannot be solved by studies focusing on single conditions or diseases [5]. For medical training, there is a need to move from more specialist to generalist medicine, and understand how to provide guidance given the intractability of creating guidelines needed for every possible set of co-occurring conditions. For health systems, there are issues of polypharmacy, misdiagnosis, mistreatment and more frequent, and possibly redundant, costly patient visits. Fundamentally, there remain serious challenges to accurate representation of what multimorbidity exists or will develop in a population [6].

The epidemiology of multimorbidity has been studied as simplified problems, with well- established methods overlooking the inter-dependencies between health conditions to focus on aggregate outcomes. The most common method of classifying individuals as multimorbid is simply counting two or more observed conditions from a predefined, non-exhaustive set of chronic conditions [7]. This approach ignores how co-occurrence of two disorders arises, which may be by chance, or due to actual shared aetiologies. It is inevitable that the more conditions considered, the more likely an individual will be classified as multimorbid. Meanwhile, factorization and dimensionality reduction have been considered to model shared underlying patient characteristics of multimorbidity [8,9]. However, dimensionality reduction diminishes the discriminative information available from condition inter-dependencies, neglecting the differences between individuals and only allowing clinicians to infer multimorbidity over a homogeneous population.

Graphs and networks can be used to represent the inter-dependencies between conditions and the overall structure of multimorbidity, retaining information that is unique to the patient or diverse populations. The simplest and most common graph construction method is to connect two conditions based on the frequency of co-occurrence [10–12]. These graphs have a strong assumption that every co-occurrence is equally important and should be considered in the wider multimorbidity graph, inferring relatedness even when conditions manifest together by chance. Graphs have also been learned using pairwise metrics, including *t*-tests, relative risk, cross entropy loss, cosine similarity and log odds [4,13–18]. However, there currently are no validated decision rules to evaluate the choice of metric or assess its suitability for different multimorbidity problems. Graphical models constitute a different class of graph learning algorithms underpinned by distributional assumptions on the data [18–22], wherein relationships between nodes are represented by probabilistic likelihoods. Graphical models can broadly be classified into two types: directed acyclic Bayesian networks and symmetric and undirected Markov networks. Generally, graphical models excel in capturing hierarchical data structures, but can be computationally expensive. As such, edges often are found through search algorithms designed to not exhaustively consider all possibilities, to reduce run time at the expense of potentially sub-optimal graphs. Despite the numerous applications of graphs in clinical studies, there is a notable lack of investigation into the quality of the multimorbidity graphs. Currently, graphs are constructed using unvalidated algorithms, often without proper thresholding and without comparison to alternative algorithms. There is an urgent need to understand hepatosplenic multimorbidity in rural, low-income settings where identification and management strategies are lacking within local health systems. Hepatosplenic multimorbidity in sub-Saharan Africa is complex and often arises due to chronic infections such as hepatitis B/C and parasitic blood flukes of *Schistosoma mansoni,* as well as concurrent alcohol use or aflatoxin exposure.

**Figure 1.**
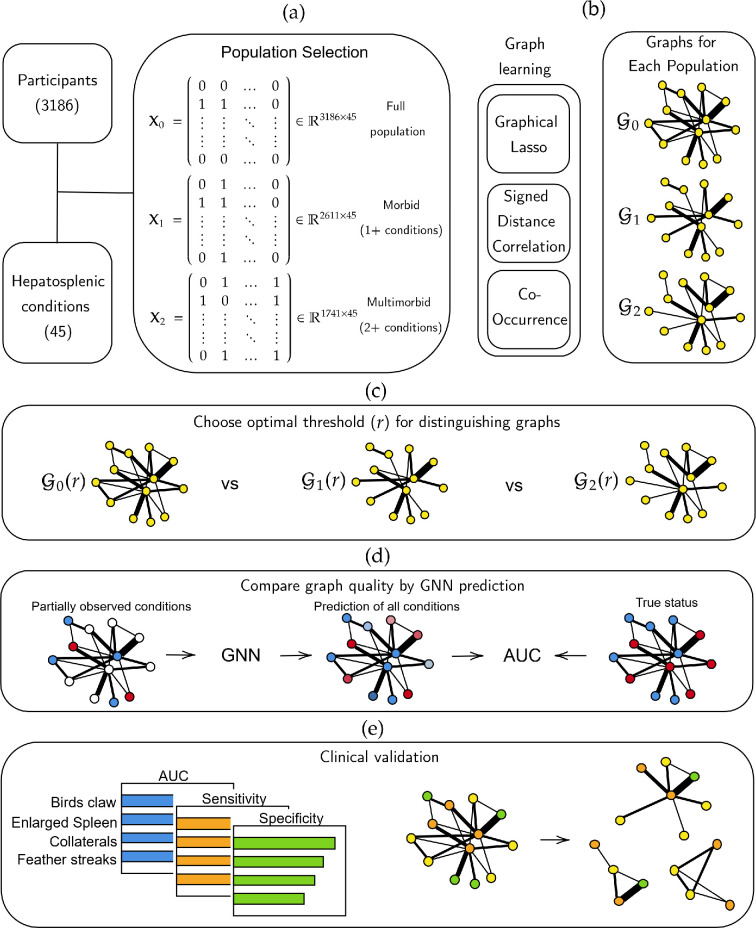
Overview of the graph learning pipeline.

In this work, we studied graphs based on real-world data, otherwise known as complex networks, applying graph-based approaches including graph neural networks and kernels to clinically validate the choice of network approach. We assessed 45 hepatosplenic conditions using point-of-care ultrasound to examine 3186 individuals in rural Uganda. We compared algorithms from three families of graph learning with different levels of statistical assumptions to represent complex hepatosplenic multimorbidity. In addition, we identified decision rules for thresholding multimorbidity graphs while accounting for the level of morbidity in the population. The quality of the graphs was evaluated based on their utility in multimorbidity prediction, and their ability to uncover insights for medical interpretation. Here, we answer the question, how can multimorbidity be assessed and validated in a manner that provides confidence for clinical decision-making? An overview of the study is presented in figure 1, and a breakdown of the conditions considered can be found in table 1.

**Table 1.** List of conditions, with the number of participants and the severity rating. *Outcomes in italics* indicate the healthy form and were not included in the multimorbidity graph. Severity rating was assigned by a sonographer, gastroenterologist and epidemiologist.

variable	outcomes	# all participants (%)	# 1+ conds (%)	# 2+ conds (%)	severity
		total = 3186	total = 2611	total = 1741	
liver patterns					
	*normal*	2340 (73.45)	1765 (67.60)	949 (54.51)	none
	unclear	15 (0.47)	15 (0.57)	15 (0.86)	mild
	feather streaks	239 (7.50)	239 (9.15)	213 (12.23)	mild
	flying saucers, starry sky	195 (6.12)	195 (7.47)	179 (10.28)	mild
	spider thickening	53 (1.66)	53 (2.03)	48 (2.76)	mild
	prominent peripheral rings	462 (14.50)	462 (17.69)	459 (26.36)	mild
	prominent pipe stems	456 (14.31)	456 (17.46)	455 (26.13)	moderate
	ruff, portal bifurcation	185 (5.81)	185 (7.09)	182 (10.45)	severe
	patches (occluded, bright white vessels)	45 (1.41)	45 (1.72)	45 (2.58)	severe
	bird’s claw	8 (0.25)	8 (0.31)	8 (0.46)	severe
other abnormalities					
	*none*	2951 (92.62)	2376 (91.00)	1525 (87.59)	none
	cirrhosis-like liver	12 (0.38)	12 (0.46)	12 (0.69)	severe
	fatty-like liver	65 (2.04)	65 (2.49)	61 (3.50)	mild
	chronic hepatitis or early cirrhosis	65 (2.04)	65 (2.49)	62 (3.56)	moderate
	polycystic kidney disease	2 (0.06)	2 (0.08)	2 (0.11)	none
	liver cysts	4 (0.13)	4 (0.15)	3 (0.17)	none
	situs inversus	1 (0.03)	1 (0.04)	1 (0.06)	none
	other	47 (1.48)	47 (1.80)	44 (2.53)	unclear
liver surface					
	*none*	3135 (98.40)	2560 (98.05)	1691 (97.13)	none
	slight/serrated	17 (0.53)	17 (0.65)	16 (0.92)	moderate
	gross/undulating	34 (1.07)	34 (1.30)	34 (1.95)	severe
caudal liver edge					
	*sharp*	2908 (91.27)	2333 (89.35)	1495 (85.87)	none
	rounded	278 (8.73)	278 (10.65)	246 (14.13)	mild
left liver lobe					
	*normal*	2212 (69.42)	1637 (62.70)	923 (53.01)	none
	moderately enlarged	428 (13.43)	428 (16.39)	364 (20.91)	mild
	moderately shrunken	357 (11.21)	357 (13.67	285 (16.37)	moderate
	enlarged	85 (2.67)	85 (3.26)	81 (4.65)	mild
	shrunken	104 (3.26)	104 (3.98)	88 (5.05)	moderate
right liver lobe					
	*normal*	2198 (68.99)	1623 (62.16)	929 (53.36)	none
	moderately enlarged	410 (12.87)	410 (15.70)	326 (18.72)	mild
	moderately shrunken	412 (12.93)	412 (15.78)	330 (18.95)	moderate
	enlarged	52 (1.63)	52 (1.99)	51 (2.93)	mild
	shrunken	114 (3.58)	114 (4.37)	105 (6.03)	severe
mean portal vein					
	*normal*	2156 (67.67)	1581 (60.55)	902 (51.81)	none
	moderately enlarged	425 (13.34)	425 (16.28)	360 (20.68)	moderate
	moderately restricted	388 (12.18)	388 (14.86)	284 (16.31)	unclear
	enlarged	168 (5.27)	168 (6.43)	154 (8.85)	mild
	restricted	49 (1.54)	49 (1.88)	41 (2.35)	unclear
portosystemic collaterals					
	*not detected*	3152 (98.93)	2577 (98.70)	1707 (98.05)	none
	splenic varices	12 (0.38)	12 (0.46)	12 (0.69)	severe
	gastro-oesophageal varices	11 (0.35)	11 (0.42)	11 (0.63)	severe
	pancreaticoduodenal varices	5 (0.16)	5 (0.19)	5 (0.29)	severe
	entirely recanalized paraumbilical vein ≥ 3 mm	1 (0.03)	1 (0.04)	1 (0.06)	severe
	splenorenal shunt	13 (0.41)	13 (0.50)	13 (0.75)	severe
	other	1 (0.03)	1 (0.04)	1 (0.06)	severe
ascites					
	*not detected*	3174 (99.62)	2599 (99.54)	1730 (99.37)	none
	yes	12 (0.38)	12 (0.46)	11 (0.63)	moderate
gall bladder visible					
	*no*	30 (0.94)	30 (1.15)	19 (1.09)	severe
	yes, but blocked by stone or collapsed	117 (3.67)	117 (4.48)	101 (5.80)	none
	*yes, clearly visible*	3090 (95.39)	2464 (94.37)	1621 (93.11)	none
gall bladder wall					
	*normal*	2939 (92.25)	2364 (90.54)	1512 (86.85)	none
	thick	247 (7.75)	247 (9.46)	229 (13.15)	severe
spleen length					
	*normal*	2188 (68.68)	1613 (61.78)	938 (53.88)	none
	moderately enlarged	387 (12.15)	387 (14.82)	315 (18.09)	moderate
	moderately shrunken	392 (12.30)	392 (15.01)	289 (16.60)	none
	enlarged	182 (5.71)	182 (6.97)	168 (9.65)	severe
	shrunken	37 (1.16)	37 (1.42)	31 (1.78)	none

## 2. Methods

### 2.1. Participants

This study was conducted within the SchistoTrack prospective cohort [23] during the first annual follow-up between 17 January and 16 February 2023. A total of 1952 households were randomly sampled from 52 villages across Buliisa, Pakwach, and Mayuge Districts of Uganda; 38 of the villages were sampled in the baseline of 2022 [24]. One child aged 5−17 years and one adult aged 18 years or older were selected by the household head or spouse and invited for clinical assessments. There were 3224 individuals clinically assessed. A total of 3186 of 3224 individuals had non-missing ultrasound data and were analyzed.

### 2.2. Hepatosplenic outcomes

We obtained hepatosplenic conditions by point-of-care ultrasound. Philips Lumify C5−2 curved linear array transducers were used with the Philips Lumify Ultrasound Application v3.0 on Lenovo 8505 F tablets with Android 9 Pie. Lossless DICOM images and videos were saved for quality assurance [24]. A number of indicators were measured including focal and diffuse liver fibrosis patterns, liver surface irregularities, caudal liver edge assessments, fatty and cirrhotic livers, liver and spleen organometry, portal vein dilation or restriction, portosystemic collaterals, ascites and gall bladder obstruction, among others. For the left and right liver lobes, spleen and portal vein diameter, we measured organometry against an internal healthy reference population standardized by height. We assessed a total of 45 hepatosplenic conditions. Detailed definitions are in the supplementary methods of the electronic supplementary material, appendix page S1.

### 2.3. Observed hepatosplenic conditions

All 45 hepatosplenic conditions were observed at least once in the study population. The most observed conditions were mildly fibrosed vessels, including prominent peripheral rings (462/3186, 14.50%) and prominent pipe stems (456/3186, 14.31%) indicative of early stage periportal fibrosis (table 1). Only 18.05% (575/3186) of participants did not exhibit any of the hepatosplenic conditions and 27.31% (870/3186) of individuals exhibited only one condition. Most of the study participants were multimorbid with 54.65% (1741/3186) of individuals with two or more conditions. The median number of conditions across all participants was two (inter-quartile range 1−3). All conditions co-occurred with another condition within at least one person. Severe conditions were observed in 22.67% (592/2611) of morbid and 30.79% (536/1741) of multimorbid individuals (table 1).

### 2.4. Population selection

While individuals who were healthy or had only one condition were often excluded from studies on multimorbidity (e.g. [10,11,16]), here, all participants were examined, and the similarity or lack thereof between graphs learned across three populations were compared including a mixed population (excluding no one), a morbid population where individuals had at least one condition and a multimorbid population where individuals had two or more conditions (the conventional population for multimorbidity studies). The splits were nested, with the mixed population fully containing the morbid, which in turn contains the multimorbid. We refer to a full population henceforth when all participants from the mixed population are used in analyses without splitting the dataset for training and testing.

### 2.5. Graph learning algorithms

We chose graph learning algorithms from three families of graphs characterized by varying levels of statistical assumptions. Analyses are performed on graphs of hepatosplenic conditions; henceforth, the conditions are referred to as nodes and the inter-dependencies between conditions as edges in the graph. Negative edges were excluded as they arose predominantly due to mutually exclusive conditions or the absence of conditions. As a baseline reference, we used co-occurrence, where edge weights were determined by the total number of individuals that exhibited both conditions concurrently. Hence, edge weights therefore depended on the size of the selected population for analysis. This method lacks any explicit statistical justification and may include edges from only one person and operates under the assumption that each additional co-occurrence contributes equally to the edge weight while ignoring chance.

As an alternative to co-occurrence, we considered the hierarchical correlation of signed distance correlation (SDC) where the coefficients were the edge weights [25]. This method combined distance correlation and Pearson correlation between two inputs as


(2.1) $$\begin{array}{lrr} & \text{sdc}(x_{i},x_{j})=\text{dcor}(x_{i},x_{j})\cdot \text{sgn}(\text{Cor}(x_{i},x_{j})). & \end{array}$$

The distance correlation was computed as


(2.2) $$\begin{array}{lrr} & \text{dcor}(x_{i},x_{j})=\frac{{\text{dist}}_{\text{cov}}(x_{i},x_{j})}{\sqrt{{\text{dist}}_{\text{cov}}(x_{i})\cdot {\text{dist}}_{\text{cov}}(x_{j})}}\in [0,1] & \end{array}$$

where $L_{2}$ norm ${\text{dist}}_{\text{cov}}(x_{i},x_{j})=\sqrt{\frac{1}{n^{2}}\sum_{n}\sum_{m}(x_{in}-x_{jm})(x_{jn}-x_{jm})}$, which allowed detection of nonlinear dependencies between the data not possible with a simple Pearson correlation. With distance correlation being restricted to positive values, the sign from Pearson correlation was introduced to identify and remove negative correlations as described in [25]. The sign of Pearson correlation equated to the Spearman when input data was binary [26], therefore, Pearson correlation was used here to remain consistent with the original methods.

To move beyond metrics for marginal associations to graphical approaches that account for the collective of variables, graphical lasso (GL) [27] was applied, which assumed the data followed a multi-variate Gaussian distribution and minimized the negative log-likelihood with a sparsity term. Compared to typical graphical models, GL has advantages in that there exist computationally efficient solutions, and integrated sparsity. The objective function consisted of


(2.3) $$\min_{\Theta }\left(-\log\det(\Theta )+\text{tr}(S\Theta )+\lambda \|\Theta {\|}_{1}\right)$$

where Θ was the precision matrix (inverse covariance) used to construct the graph, −log⁡det(Θ)+tr(SΘ) came from the multi-variate Gaussian negative log-likelihood with sample empirical covariance S. The $\|\Theta {\|}_{1}$ term induced sparsity by penalizing the magnitude of the entries in Θ, while λ was the tuning parameter that controlled the trade-off between the log-likelihood and the sparsity term selected through fivefold cross-validation using the GraphLassoCV package from sklearn 1.5.0 in Python 3.9.

### 2.6. Thresholding via maximizing graph dissimilarity

Thresholding was applied to remove weak connections that may be uninformative and potentially influenced by noise in the data. The algorithms we considered produced different magnitudes of edge weights, therefore percentage thresholding was used to determine the appropriate cutoff. We determined the best threshold by maximizing the structural difference between graphs generated on the three populations used to represent multimorbidity. Graph kernels were used to measure structural similarity between two graphs [28], where the lower the value the more different the two graphs. We considered three graph kernels from the GraKeL library v0.1.10 in Python 3.9, Weisfeiler-Lehman [29], Subgraph matching [30] and Neighbourhood hash [31]; details can be found in electronic supplementary material, appendix page S5.

We measured the similarity between graphs learned on every combination of the three populations. Kernels were computed over graphs constructed from 500 random samples (to obtain standard deviations) where each sample consisted of a uniform probability random sample of 50% of the study population. The optimal threshold was the location of the minimum kernel value. We averaged over the three kernels and across the three possible population comparisons. Each threshold from each algorithm was applied to the sample graphs to obtain the thresholded graphs, as well as the final graphs produced from each algorithm when re-learned over all participants.

### 2.7. Algorithm comparison via predictive modelling with GNNs

To evaluate the quality of the graphs from each algorithm, we utilized graph neural networks (GNNs) for the task of multimorbidity prediction. Given the observed status of m conditions in an individual, we predicted the status of the full set of 45 conditions assessed in this study. This problem could be viewed as utilizing a partially observed graph representing a scenario of when not all conditions are diagnosed in an individual. We only considered 10 training splits in this experiment, using the first 10 seeds from the previous experiment. A total of 50% of participants were used to train the GNN and the remaining 50% were held out as test sets. For each split, we randomly selected a subset of m conditions to predict both the set of observed conditions and a wider set of unobserved conditions. This approach was taken to represent the problem of where some conditions are known to occur in a population, yet there is a need to predict statuses in new patients. The status of each condition was binary, making this a vectorial binary classification over the 45 conditions, and we evaluated performance by AUC, sensitivity and specificity. All GNNs were set to two layers and a fixed width to allow for comparison across GNNs. Three architectures of graph convolutional network (GCN) [32], graph attention network (GAT) [33] and sample and aggregate (GraphSAGE) [34] were chosen based on their spatial usage of the graph and ease of interpretability. Details can be found in electronic supplementary material, appendix page S5. We applied the GNNs to the thresholded graphs with the number of condition inputs m=5,10,…,25. In this experiment, we also present predictions for only unobserved conditions to represent when new diagnoses need to be evaluated within a patient. As validation analyses, we also varied the threshold to test and compare against the optimal thresholds, and, fixing m=5, applied the GNNs to populations with different levels of morbidity (full population, morbid and multimorbid), to examine the effect of excluding individuals from the study population.

### 2.8. Clinical validation

An infectious disease epidemiologist (GFC), sonographer (SM) and gastroenterologist (CKO) independently ranked the risk of each condition for schistosomal portal hypertension as none/unclear, mild, moderate or severe. The majority vote was taken to classify conditions, and the clinical utility of graph structures and GNN performance was assessed by condition severity. AUC, sensitivity, and specificity were calculated for each condition averaged over the 10 splits with a random selection of five input conditions. For sensitivity and specificity, a universal cut-off was selected based on the highest of the two quantities combined over the 45 conditions. Global graph properties were examined to assess improvements in sparsity between thresholded and non-thresholded graphs as well as to assess graph stability for clinical decision-making.

## 3. Results

### 3.1. Learned graphs and thresholds

**Figure 2.**
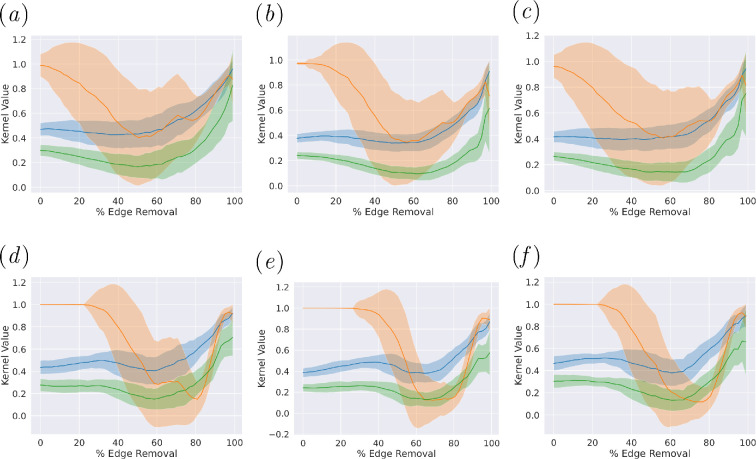
Graph kernels results. Blue: Weisfeiler Lehman, Orange: Subgraph Matching, Green: Neighbourhood Hash. (*a*) GL full vs. morbid. (*b*) GL full vs. multimorbid. (*c*) GL morbid vs. multimorbid. (*d*) Signed-distance correlation (SDC) full vs. morbid. (*e*) SDC full vs multimorbid. (*f*) SDC morbid vs multimorbid. Plots show the similarity measures between graphs learned the full population, morbid (1+ condition), and multi-morbid people (2+ conditions) using graph kernels. This experiment cannot be applied to co-occurrence graphs as they are identical when constructed from any of the three populations.

GL and SDC both consistently produced the strongest edge between prominent peripheral rings and prominent pipe stems. These conditions were two different cross-section views of the same pathology and the most observed co-occurrence with a frequency of 437 of 3186 participants. Other regularly observed edges across the samples for each algorithm and population are in electronic supplementary material, appendix figures S1, S2, and S3. Figure 2 presents the thresholding analysis using graph kernels. The final thresholds were 50.16% for GL and 64.46% for SDC.

**Figure 3.**
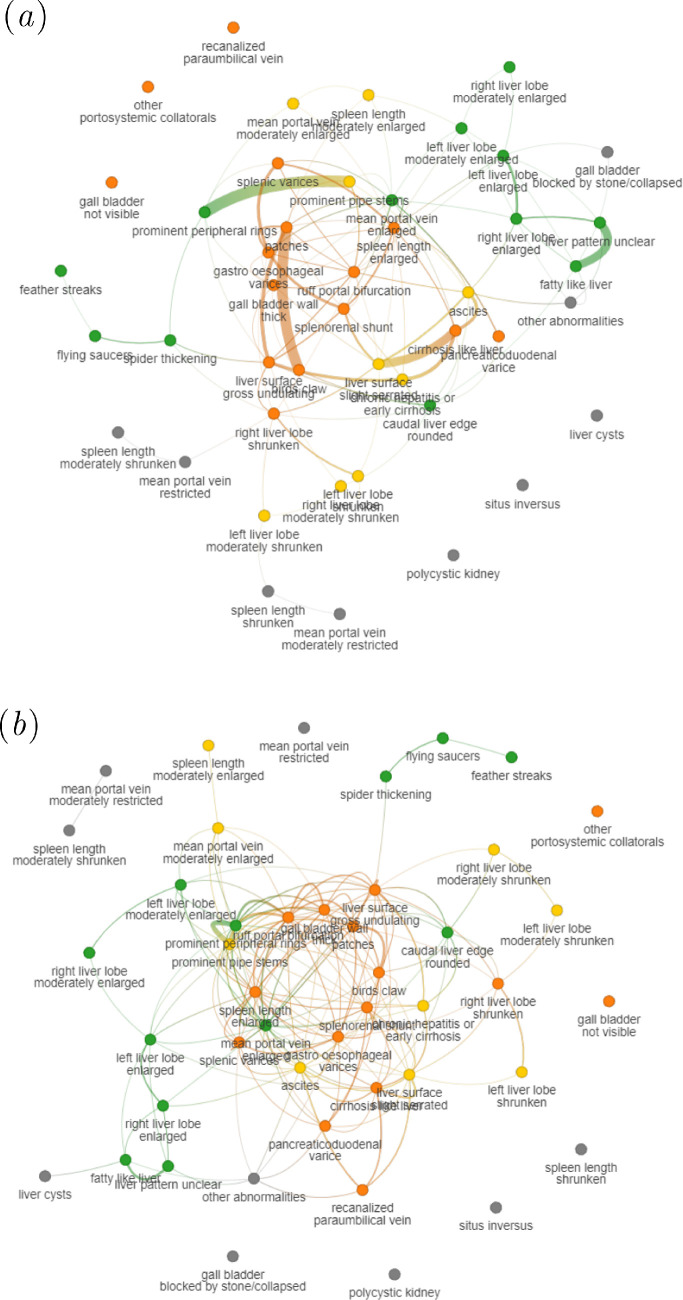
Final graphs using optimal percentage thresholds coloured by severity. Each graph is learned on the full dataset using the average thresholds found from the graph kernels experiment for (*a*) GL (50.16%) and (*b*) SDC (64.46%). Nodes are coloured by the severity of the condition, rated by a sonographer, gastroenterologist and epidemiologist, with respect to risk of schistosomal portal hypertension where green is mild; yellow is moderate; orange is severe; grey is none/unclear.

Global properties of the 500 thresholded graphs are presented in table 2 (non-thresholded graphs in electronic supplementary material, appendix table S1). Despite a higher percentage threshold, SDC was denser than GL, and had a more discernible degree structure fitting to a log-normal distribution while GL did not fit common degree distributions. SDC and GL had edge densities of 0.17 and 0.11 with average degrees of 7.29 and 4.84, respectively. There was a 54% overlap in edges between the two graphs when considering the union of edges. GL maintained similar global properties between the final graph and the training samples, whereas SDC was sensitive to the sample size, and the final and training samples from SDC were significantly different in a number of statistics. Concerning co-occurrence, there were no identifiable thresholds as co-occurrence edges were only defined by multimorbid participants and excluding the healthy and morbid did not change the graph. Consequently, co-occurrence had the highest edge density of 0.55 and average degree of 24.23 (exhibiting a power law degree distribution) where all nodes were connected in a single component (electronic supplementary material, appendix table S1). The final thresholded graphs computed over all participants are shown in figure 3, whereas the unthresholded co-occurrence graph is presented in electronic supplementary material, appendix figure S6 along with the unthresholded GL (electronic supplementary material, appendix figure S4) and SDC (electronic supplementary material, appendix figure S5).

**Table 2.** Sample and final graph statistics. Sample graph statistics were computed over 500 samples at optimal thresholds, and final graphs were learned on training and testing sets combined. Final GL and SDC graphs share 92 edges, and 34 nodes in their largest components. Degree distribution of none indicated that the graphs did not fit any of exponential, power law or log-normal.

	graphical lasso samples	final GL graph	signed distance correlation samples	final SDC graph
number of edges	110.99 ± 4.60	109	148.37 ± 4.84	164
average degree	4.93 ± 0.20	4.84	6.59 ± 0.22	7.29
average clustering coefficient	0.21 ± 0.03	0.26	0.37 ± 0.03	0.39
diameter	6.30 ± 0.93	6	6.23 ± 0.99	7
edge density	0.11 ± 0.00	0.11	0.15 ± 0.00	0.17
largest component size	39.64 ± 1.65	39	36.37 ± 1.81	36
number of isolated nodes	4.73 ± 1.27	6	6.83 ± 1.63	7
assortativity	0.12 ± 0.08	0.12	0.25 ± 0.07	0.32
largest component spectral gap	0.66 ± 0.30	0.32	0.04 ± 0.04	0.03
degree distribution	none	none	log-normal	log-normal

### 3.2. Graph quality evaluation by GNN predictive analysis

**Figure 4.**
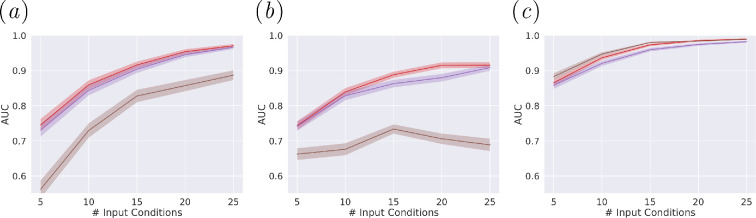
Multimorbidity prediction with varying number of inputs. (*a*) GCN, (*b*) GAT and (*c*) GraphSAGE, the model uses graphs with optimal thresholds, Red: GL 50.16%, Purple: SDC 64.46%, Brown: Co-occurrence 0%. An optimal threshold could not be found for the co-occurrence graph, so 0 threshold was used instead; this matched with what had been done in the literature [10,11]. All AUCs were averaged over 10 training-testing splits.

Figure 4 compares the thresholded graphs and GNNs used for multimorbidity prediction (further validation by varying thresholds shown in electronic supplementary material, appendix figures S8–S18). The prediction problem, which used a partially observed graph, represented a scenario for when only some conditions are observed in a patient and there is a need to also predict undiagnosed conditions. For example, when only 11% (5/45) of conditions were observed by the GCN, the average AUC was 0.75, 0.73 and 0.56 for GL, SDC and co-occurrence, respectively. Out of the 10 training splits, the highest test AUC (0.78) was achieved when indicators of liver fibrosis, fatty livers, portal vein enlargement possibility indicative of portal hypertension and hypersplenism were observed. The worst performing split included rounded liver edges, recanalized paraumbilical veins, gall bladder injuries and abnormal spleen organometry (AUC of 0.69). GL and SDC performed similarly if not better when AUCs were evaluated over only unobserved conditions (see electronic supplementary material, appendix figure S7). Generally, GL and SDC produced similar AUCs with all three GNNs. Although marginally, GL performed best when considering consistent differences to SDC across varying numbers of condition inputs for GAT and GraphSAGE. Co-occurrence, on the other hand, with 0% thresholding, was the worst performing graph when evaluated with GCN and GAT. The dense nature of the co-occurrence graph was better handled with GraphSAGE due to its sampling element, the model inherently used subsets of each neighbourhoods in the model aggregation, leading to comparable performances between all three graphs. Similar results on unobserved conditions only are presented in electronic supplementary material, appendix figure S7. All three GNNs performed similarly for GL and SDC when tested over each of the mixed, morbid and multimorbid populations; whereas the co-occurrence graph construction was only possible for a multimorbid population (see electronic supplementary material, appendix figures S9−S18).

### 3.3. Hepatosplenic multimorbidity

**Figure 5.**
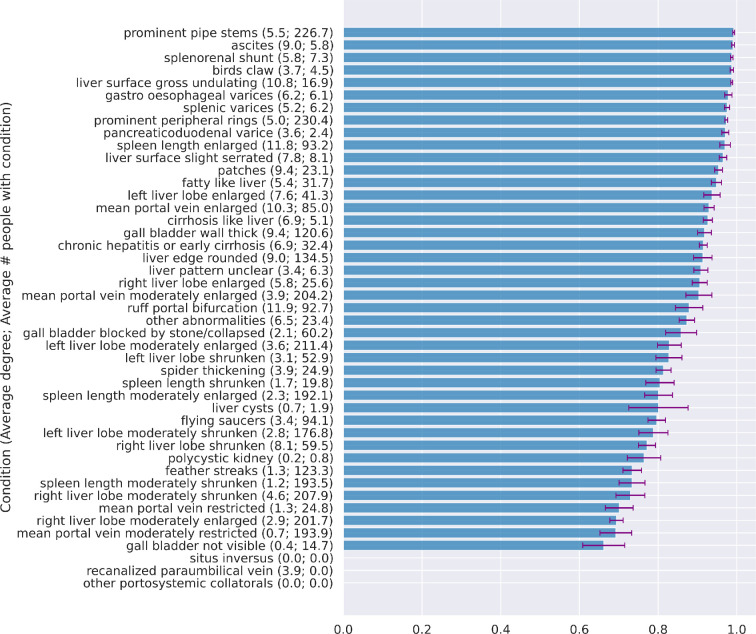
Performance on each condition ordered by AUC. Results were produced by a GCN using GL at optimal threshold on the test sets. Results on SDC and co-occurrence can be found in electronic supplementary material, appendix figures S19, S20, and S21. For conditions only observed once, the positive patients were required to be in the training set to run the graph learning algorithms, so the testing set consisted of only one class and AUCs did not exist. For these conditions one can observe the performances on the training sets from the additional results in electronic supplementary material, appendix figures S19, S20, and S21 for an informed indication of performance.

**Figure 6.**
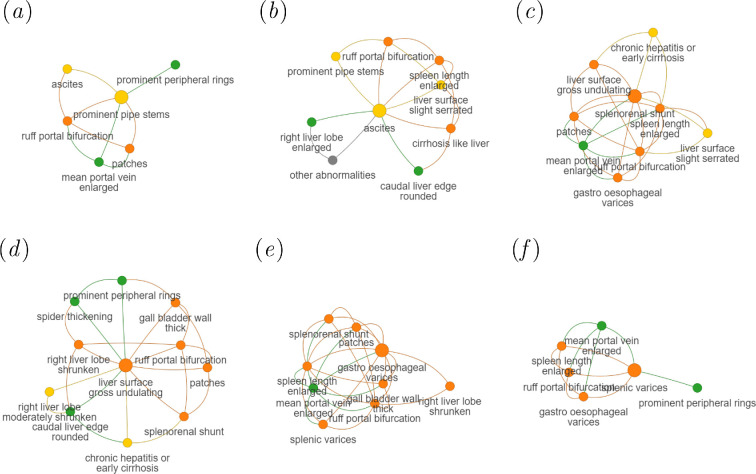
Neighbourhood subgraphs of the best predicted conditions. Examples are taken from figure 5. The subgraphs are taken from thresholded final GL graph. (*a*) Prominent pipe stems, AUC = 0.992 ± 0.007. (*b*) Ascites, AUC = 0.990 ± 0.011. (*c*) Splenorenal shunt, AUC = 0.987 ± 0.010. (*d*) Liver surface gross undulating, AUC = 0.987 ± 0.007. (*e*) Gastro oesophageal varices, AUC = 0.978 ± 0.030. (*f*) Splenic variaces, AUC = 0.975 ± 0.021. Nodes are coloured by the severity of the condition, rated by a sonographer, gastroenterologist and epidemiologist, with respect to risk of schistosomal portal hypertension where green is mild; yellow is moderate; orange is severe; grey is none/unclear.

Figure 5 presents the GCN model performance broken down for each condition using GL due to the marginally superior performance when compared to SDC. For conditions not exhibited by anyone in the test set, AUC cannot be computed, so we observed the training set for an indication of their performances (electronic supplementary material, appendix figures S19, S20, and S21). With AUCs > 0.99, the conditions best predicted most often were severe conditions related to a high risk of schistosomal portal hypertension. The final graphs from GL and SDC are presented in figure 3*a*,*b*. Severe conditions were concentrated in the core of the final graphs with the highest degrees, whereas mild or irrelevant conditions for schistosomal portal hypertension were on the periphery. The frequency of each severe condition vastly varied. Sensitivity and specificity for each condition are shown in electronic supplementary material, appendix figures S22 and S25 (more detail is provided in electronic supplementary material, appendix figures S22–S27). Using GL, the GCN model showed higher sensitivity than specificity for 88% (37/42) of conditions (three conditions could not be evaluated) with an average sensitivity of 0.91 (s.d. 0.15) and average specificity of 0.67 (s.d. 0.25). The GCN model predicted the majority of severe conditions with perfect sensitivity (median = 1) and high specificity (median > 0.74). One-hop neighbourhood subgraphs for moderate and severe conditions from GL are shown in figure 6. For example, conditions considered multimorbid (or likely to develop) with the liver condition of prominent pipe stems, which was predicted with high accuracy (AUC 0.992), included ascites, patches, enlarged mean portal vein and ruff portal bifurcation. Irrespective of whether GL or SDC were used with GCNs, there was a clear trend in better performance for the prediction of severe conditions as compared to moderate, mild or irrelevant conditions for schistosomal portal hypertension (see electronic supplementary material, appendix figures S28 and S29).

## 4. Discussion

Graph learning is an essential step towards understanding complex multimorbidity. A total of 3186 individuals within the SchistoTrack study in rural Uganda were diagnosed with 45 hepatosplenic conditions using point-of-care ultrasound. Analyzing these conditions, we presented a machine learning pipeline to learn clinically useful graphs. We established decision rules for thresholding statistically relevant condition inter-dependencies and evaluated the graphs for multimorbidity prediction. We showed that co-occurrence graphs were poorly suited for problems of multimorbidity. Our study produced sparser, more interpretable graphs using GL and SDC that offered clinical insights for understanding hepatosplenic morbidity in low-income countries.

While co-occurrence has been regularly used to construct multimorbidity graphs [10–12], we found co-occurrence graphs to be over-specified (overestimating multimorbidity), dense with little discriminatory information for conditions, and with low predictive utility. Sparser graphs than those observable with co-occurrence enable superior predictive modelling and are easier to interpret [35]. We showed that thresholding graphs based on maximizing structural differences between full, morbid and multimorbid populations removed weak inter-dependencies (edges) that represented insignificant relations or noise in the data, producing clinically informative sparse multimorbidity graphs. In particular, GL had the lowest edge density and average degree when compared to co-occurrence or SDC. Both GL and SDC detected nodes as isolates, indicating the lack of significant clinical associations with other conditions, while co-occurrence forced inter-dependencies between these conditions that co-existed in a small number of people (often just one), which is insufficient evidence to identify any aspect of multimorbidity as a public health problem across a population.

The limited utility of co-occurrence was further evident when applied to the task of multimorbidity prediction. Graphs from GL and SDC both improved neural network models by similar margins, and significantly better than co-occurrence in two of the three GNNs. A near complete removal of all graph edges led to the lowest AUCs from each GNN, highlighting the utility of the graphs. These results indicate that multimorbidity could be represented accurately by multiple graphs from inherently different algorithms that have at least some level of statistical assumption. Although co-occurrence performed well with GraphSAGE, GraphSAGE has a sampling framework that is well-suited to denser graphs, as only a subset of neighbours were used from each node. Although GraphSAGE could potentially alleviate the need for thresholding, this model is less interpretable clinically as the stochasticity makes it difficult to identify inter-dependencies for a diagnosed condition, whereas conditions might not be included in the aggregation steps of GraphSAGE despite belonging to the neighbourhood of the condition of interest. Thus, despite the better performance of GraphSAGE [11], when building models for predictions with good interpretability, GCN and GAT were better supported here for multimorbidity problems.

Methods of analyzing graphs in order to define multimorbidity vary widely. Community detection often is performed on graphs, with each cluster of conditions labelled as multimorbid [5,9,10]. Edge prediction has been explored and multimorbidity has been conceptualized as pairwise relationships between two conditions of interest [36]. Here, we proposed a different approach to understanding multimorbidity that takes into account the entire complex system of hepatosplenic conditions. We characterized multimorbidity by the diagnosis and prediction of multiple conditions, extending far beyond the prediction of two conditions and retaining information specific to each individual condition. The practical usage of these graphs may be as follows. If a patient was diagnosed with one condition, the sub-graph centred on that condition could be inspected to identify what the patient also is likely to have or what later conditions they are likely to develop. For example, our study suggests that individuals with mild periportal fibrosis might later develop severe conditions indicative of portal hypertension such as ascites, extensive portal fibrosis and enlarged main portal vein diameters. In the case of multiple positive diagnoses, a clinician may consider using the union of neighbourhoods in our graphs to derive the full set of multimorbidity in a patient. Remarkably, we found that severe conditions were central to the validated graph structures, exhibiting the most complex multimorbidity.

The GCN had the most interpretable architecture and performed marginally better with GL, therefore it was analyzed to reveal the best and worst predicted conditions. Conditions with very few degrees or isolate conditions generally had the lowest AUCs. Hence, connectivity was important for modelling multimorbidity and the use of neighbourhood information supported more accurate predictions of undiagnosed conditions. Interestingly, the best predicted conditions were not always the most connected (hubs) nor necessarily the most frequent (highest population prevalence), but were the most severe conditions that confer the highest risk of life-threatening portal hypertension. Overall, when focusing on the prediction of individual conditions, our break down of the GCN model showed it was much better at predicting positive diagnoses rather than the absence of a diagnosis or condition (higher sensitivity than specificity). This trend is to be expected as in clinical practice, with limited time and resources, it often is an insurmountable problem to confirm true negatives without extensive, exhaustive alternative diagnostics such as alternative imaging modalities or biopsies that would have been needed here. Thus, if using our model for hepatosplenic conditions, one may envision confidence in providing treatments or at the very least informing triage when a patient is predicted to have a set of conditions—especially that require urgent medical care—but we would recommend further clinical review and follow-up if a patient is predicted as unlikely to have a set of conditions. All of which is in line with standard general medicine practice.

Learning how to accurately represent multimorbidity using graph learning for clinical-decision-making opens avenues for more advanced modelling of multimorbidity that incorporates individual patient characteristics into multi-output models (e.g. [37–39]). There is a need to move current medical practice beyond focusing on one disease per patient. The pipeline proposed, if replicated and generalised in future studies on other diseases and populations, could help progress towards a better understanding of multimorbidity. Our work not only revealed the complex system of inter-dependencies for hepatosplenic conditions, but also provides a validated machine learning pipeline for clinical and research communities to understand multimorbidity as a public health problem.

## Data Availability

The model pipeline code is shared along with the relevant data to run the pipeline, focusing on the three network edge lists, as supplementary material [40]. Individual participant data for the predictions could not be shared due to their identifiable nature and associated ethics restrictions, data privacy considerations and the ongoing nature of the SchistoTrack cohort. Dummy data are provided for GNN predictions reliant on individual-level data where graphs can be loaded using pre-computed edge lists in place of learning the graph.
